# Formin protein FMNL1 is a biomarker for tumor-infiltrating immune cells and associated with well immunotherapeutic response

**DOI:** 10.7150/jca.86965

**Published:** 2023-09-11

**Authors:** Guomin Lu, Hui Wang, Rui Xu, Junying Xu, Fangmei An, Haoran Xu, He Nie, Jie Mei, Qiang Zhan, Qinglin Zhang

**Affiliations:** 1 Departments of Gastroenterology, The Affiliated Wuxi People's Hospital of Nanjing Medical University, Wuxi Medical Center, Nanjing Medical University, Wuxi, Jiangsu 214023, China.; 2 The First College of Clinical Medicine, Nanjing Medical University, Nanjing, Jiangsu 211166, China.; 3 Departments of Oncology, The Affiliated Wuxi People's Hospital of Nanjing Medical University, Wuxi Medical Center, Nanjing Medical University, Wuxi, Jiangsu 214023, China.

**Keywords:** FMNL1, bioinformatics, biomarker, cancer, mQIF

## Abstract

**Background:** Increased studies on the basis of bulk RNA-sequencing (RNA-seq) data of cancer identify numbers of immune-related genes which may play potential regulatory roles in the tumor microenvironment (TME) without in-depth validation.

**Methods:** In the current study, the immunological correlation and cell subpopulation expression pattern of FMNL1 were analyzed using public data. In addition, the cell subpopulation expression pattern of FMNL1 was also deeply validated using single-cell RNA-sequencing (scRNA-seq) and multiplexed quantitative immunofluorescence (mQIF).

**Results:** Bulk *FMNL1* mRNA was related to better prognosis in hepatocellular carcinoma (HCC) and was able to identify immuno-hot tumor in not only HCC but also multiple cancer types. Bulk *FMNL1* mRNA also predicted the response to immunotherapy in multiple cancers. Further validation using scRNA-seq and mQIF revealed that FMNL1 was a biomarker for immune cells.

**Conclusions:** FMNL1 is a biomarker for immune cells in not only hepatocellular carcinoma, but also multiple cancer types. Moreover, immune infiltration analysis using the bulk RNA-seq data would be further validated using scRNA-seq and/or mQIF to describe the cell subpopulation expression pattern in tumor tissues for more in-depth and appropriate understanding.

## Background

Increasing studies on the basis of bulk RNA-sequencing (RNA-seq) data of cancer have been conducted to investigate the features of the tumor microenvironment (TME) and identify numbers of immune-related genes that may play potential regulatory roles in TME, but only a few researchers provided in-depth insights into candidate genes 1, 2. In fact, most immune-related genes are lowly expressed in tumor cells but specifically enhanced in immune cells. In other words, these genes were just novel biomarkers for immune cells, but not cancer immunological correlation genes. For example, PD-L1 is induced by IFN-γ released by cytotoxic T lymphocytes (CTLs) and promotes the immune escape of cancer cells. The expression of PD-L1 is highly correlated with the TME features. However, CD8A, a cell biomarker for CTLs, is also highly correlated with TME features in bulk RNA-seq analysis. Obviously, the tight immunological correlation of CD8A makes no sense for cancer cell-mediated TME regulation because it is just a cell biomarker for CTLs. However, bulk RNA-seq fails to resolve cell subpopulations in tumor tissues 3, and can not effectively identify the function of candidate genes.

In our previous study, we uncovered that Formin-like gene 1 (FMNL1) was tightly related to immune infiltration in gastric cancer 4. FMNL1 is highly expressed in leukocytes and overexpressed in lymphomas, but also expresses in epithelial cancer 5, 6. In terms of molecular functions, FMNL1 is a classical cytoskeleton regulator characterized by the FH2 structural domain, which mediates the assembly of actin filaments 7. Consequently, FMNL1 has been summarized to be correlated with the aggressiveness of multiple cancers 8, 9. Behind the tight immunological correlation, whether FMNL1 plays a critical role in regulating TME or it is just a biomarker for tumor-infiltrating immune cell (TIIC) should be further investigated.

In this research, the bulk RNA-seq data from the Cancer Genome Atlas (TCGA) database was utilized to explore the correlation between FMNL1 and features of TME, and FMNL1 was found to be tightly correlated with features of TME in not only hepatocellular carcinoma (HCC) but also multiple cancer types. However, further validation using single-cell RNA-sequencing (scRNA-seq) and multiplexed quantitative immunofluorescence (mQIF) revealed that, FMNL1 was just a biomarker for immune cells. Thus, bulk RNA-seq analysis of immune-related genes should be further validated using sc-RNA-seq or mQIF to describe the cell subpopulation expression pattern in tumor tissues.

## Materials and methods

### Acquisition of public transcriptome data

The RNA-seq data and clinical annotation in the Cancer Genome Atlas (TCGA) were obtained from the Xena (http://xenabrowser.net/datapages/). All abbreviations for tumor types were shown in Table S1. The single-cell RNA sequencing (scRNA-seq) datasets (GSE98638, GSE125449, and GSE140228) from hepatocellular carcinoma (HCC) patients were collected from previously published datasets 10-12.

### Assessment of immunological characteristics of the tumor microenvironment

The immunological features of the tumor microenvironment (TME) in HCC contained immunomodulators, the activities of the cancer immunity cycle, infiltration levels of tumor-infiltrating immune cells (TIICs), and the expressions of inhibitory immune checkpoints. First, the ESTIMATE algorithm was apllied to calculate Tumor Purity, ESTIMATE Score, Immune Score, and Stromal Score 13. In addition, we studied the expressions of 122 immune modulators, including MHC, receptors, chemokines, and immuno-stimulating factors 14. Furthermore, the correlations between FMNL1 expression and immune checkpoints levels were assessed. We used five independent algorithms to calculate TIICs levels to prevent calculation errors brought on by different techniques: TIMER 15 EPIC 16, MCP-counter 17, quanTiseq 18, and TISIDB 19. The single-sample gene sets enrichment analysis (ssGSEA) 20 was used to estimate the infiltration levels of various immune cell populations as well as the activity of immune-related pathways and functions of each patient in order to better understand the immunological status of each patient using a set of signature genes of 29 immune cell types and immune-related pathways 21. We also calculated the T cell inflamed score using the weighting coefficient and expression levels of 18 genes 22. Given that the anti-cancer immune response was mirrored by a cancer immunity cycle with seven phases, the activation score of each stage was determined by the expression of particular biomarkers in each stage by ssGSEA 20. According to previous studies 23-26, immuno-hot tumors could be defined as tumors with high expression immunomodulators, high activities of the cancer immunity cycle, high levels of TIICs, and high expression of inhibitory immune checkpoints. We split the patients into high and low FMNL1 groups at the median expression of FMNL1 in order to explore the function of FMNL1 in controlling anti-tumor immunity in HCC. We next analyzed the differences in immunological characteristics of TME in these aspects.

### Single-cell RNA sequencing data analysis

The standard workflow of the python-based toolkit Scanpy (version 1.5.1) 27 was used to carry out unsupervised clustering and dimension reduction. In short, highly variable genes (HVGs) were found utilizing dispersion-based approaches by setting flavor = “seurat”, where the normalized dispersion is created by scaling with the mean and standard deviation of the dispersions for genes falling into a specific bin for mean expression of genes. The variable gene matrix underwent Principal Component Analysis (PCA) to decrease noise, and the top 50 PCs were used in subsequent analyses. A batch-balance k nearest neighbor (KNN) graph was created using BBKNN to correct the technical batch effects from heterogeneous datasets by identifying the top neighbors of each cell in each batch independently as opposed to the full cell pool 28. The Leiden technique was then used on these nearest neighbor graphs to identify cell clusters and identify communities 29. The same PCs recommended using uniform manifold approximation and projection (UMAP) for visualization 30. In earlier investigations, the cell type for every cell was annotated.

The scRNA-seq database Tumor Immune Single-cell Hub (TISCH, http://tisch.comp-genomics.org/gallery/) 31, which focuses on the exploration of the TME features, offers thorough single-cell level cell-type annotation. The cell subpopulation patterns of FMNL1 in human malignancies were examined using the TISCH. All settings were set to their default values.

### Cell-cell communication analysis

Cell-cell communications mediated by ligand-receptor complexes were critical to diverse biological processes, such as inflammation and tumorigenesis. To investigate the molecular interaction networks between different cell types, we used the “CellPhoneDB” 32, a software to infer cell-cell communication from the combined expression of multi-subunit ligand-receptor complexes, to analyze the interactions between tumor cells and microenvironment cell subpopulations. The ligand-receptor pairs with a P value < 0.05 were remained for the assessment of relationship among different cell clusters.

### Clinical samples

The HCC tissue microarray (Cat. HLivH180Su17) was provided by Outdo BioTech (Shanghai, China). The tissue microarray included 88 para-tumor tissues and 92 HCC tissues. Outdo BioTech provided comprehensive clinico-pathological details and follow-up information for the cohorts. In this study, the tissue microarray was used for multiplexed quantitative immunofluorescence (mQIF). The Clinical Research Ethics Committee of Outdo Biotech (Shanghai, China) gave its approval for the research of tissue microarray.

### Multiplexed quantitative immunofluorescence

Using a previously established procedure, the mQIF was immediately carried out on the tissue microarray to quantify the levels of FMNL1, CD45, and CK8 in the HCC samples and simultaneously detecting DAPI 33. The primary antibodies were as follows: anti‑FMNL1 (1:4000 dilution, Cat. 27834-1-AP, ProteinTech, Wuhan, China), anti‑CD45 (1:10 dilution, Cat. GM0701, GeneTech, Shanghai, China), and anti‑CK8 (1:5 dilution, Cat. GT2035, GeneTech, Shanghai, China). For stratification, samples were classified into high/low at the 50-percentile of the cohort scores as stratification cut-point. CK8 staining was used to distinguish the tumor and stromal regions. Using HistoQuest software (TissueGnostics), positive/negative cells were identified in 3-5 typical locations (0.23 mm2 each). In the representative locations, the rates of positive cells were calculated.

### Predictive value of FMNL1 in the response to immunotherapy

To check whether the bulk *FMNL1* mRNA level could predict the response to immunotherapy, the GSE100797, GSE126044, MEDI4736, and PRJEB23709 cohorts 34-37 were downloaded. The expression of FMNL1 in samples with different responses was compared. In addition, the prognostic value of FMNL1 in the PRJEB23079 cohort was assessed.

### Human Protein Atlas database analysis

The Human Protein Atlas (http://www.proteinatlas.org/) dataset is launched to describe all the human proteins in cells, tissues, and organs utilizing the integration of multi-omics 38. All researchers can obtain the online data for free on the HPA platform. In this study, the protein levels of FMNL1 in tumor tissues across cancer types were examined using data programmed from the HPA dataset.

### Statistical analysis

R version 4.0.0 was used to conduct all statistical analyses. The difference between two groups was assessed using the parametric Student's t-test or non-parametric Mann Whitney test, and the difference between several groups was examined using the parametric one-way ANOVA or the non-parametric Kruskal-Wallis test. The log-rank test was used for the survival analysis. The Pearson test was used to examine the correlation between the two variables. If not otherwise specified, a two-paired P-value < 0.05 was considered statistically significant for all analyses.

## Results

### Bulk *FMNL1* mRNA expression predicts better prognosis in HCC

**Figure 1:**
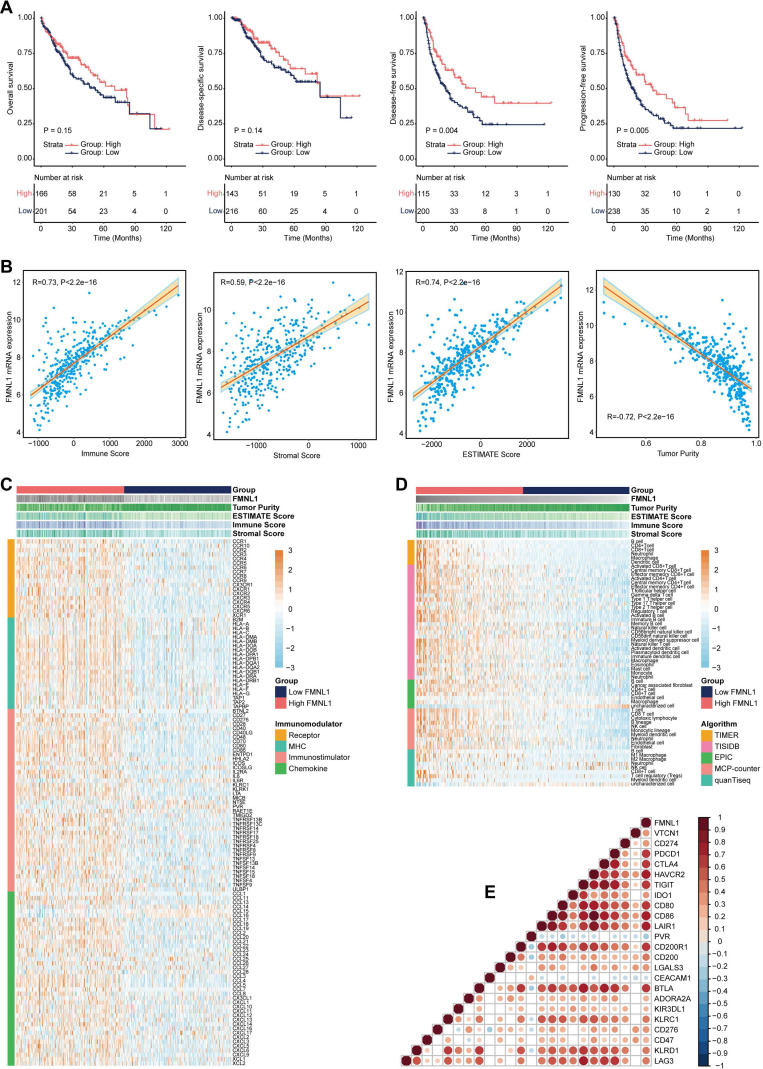
** FMNL1 identifies the immuno-hot TME and better prognosis in the TCGA-LIHC cohort.** (A) Prognostic values of *FMNL1* mRNA in HCC in terms of OS, DSS, DFS, and PFS. (B) Correlations between *FMNL1* mRNA expression and Tumor Purity, ESTIMATE Score, Immune Score and Stromal Score estimated by ESTIMATE algorithm in HCC. (C) Expression levels of 122 immunomodulators in HCC between the high and low FMNL1 groups in HCC. (D) The difference of TIICs levels between high and low FMNL1 groups calculated by five independent algorithms in HCC. (E) Correlations between FMNL1 and inhibitory immune checkpoints in HCC. The color reveals the Pearson correlation coefficient.

The clinical parameters of the TCGA-HCC cohort were exhibited in Supplementary Table S2. Firstly, the prognostic value of *FMNL1* mRNA was assessed in HCC. As Figure 1A showed that patients with high *FMNL1* mRNA expression exhibited prolonged disease-free survival (DFS) and progression-free survival (PFS), but had no significant correlation with overall survival (OS) and disease-specific survival (DSS). We also examined the correlation between FMNL1 expression and clinical parameters, and found that FMNL1 was correlated with gender (Supplementary Table S3). Given the oncogenic role of FMNL1 in human cancers 8, 9, the contradictory prognostic phenotype was puzzling. Generally, patients with immuno-hot tumors exhibited a better prognosis 39. Based on previous research 4, we speculated that FMNL1 was correlated with immuno-hot tumors in HCC, and then the immunological role of FMNL1 was investigated in the TCGA cohort.

### Bulk *FMNL1* mRNA expression identifies immuno-hot tumors in HCC

According to the results of the ESTIMATE algorithm in HCC, tumors with high FMNL1 expression had lower tumor purity but higher TIIC infiltration (Figure 1B). Bulk FMNL1 mRNA expression was positively correlated with ESTIMATE Score, Immune Score, and Stromal Score but negatively correlated with Tumor Purity. The majority of chemokines, immunostimulators, major histocompatibility complex (MHC) components, and receptors were overexpressed in the high FMNL1 group, according to further expression level analysis (Figure 1C). In addition, we assessed the gene biomarkers of common immune cells and found that these biomarkers were upregulated in the high FMNL1 group (Supplementary Figure S1A). Subsequently, the infiltration levels of TIICs were estimated using five independent algorithms, and the results revealed that the infiltration levels of most TIICs were significantly upregulated in the high FMNL1 group (Figure 1D). Simultaneously, we discovered that FMNL1 was positively linked with the majority of immune cell types and the activity of immune-related pathways except type II IFN response (Supplementary Figure S1B), by utilizing the ssGSEA algorithm. FMNL1 expression was positively correlated with the T-cell inflamed score, an alternative indicator to evaluate the clinical response to immunotherapy, and the activities associated with most steps of cancer immunity cycle were significantly upregulated in the high FMNL1 group (Supplementary Figure S1C-D). More meaningfully, FMNL1 in HCC was found to be positively correlated with most inhibitory immune checkpoints, which were uncovered to be highly expressed in the inflamed TME (Figure 1E). Overall, *FMNL1* mRNA is highly correlated with the immuno-hot TME in HCC and could be used as a novel biomarker to predict better prognosis.

### Pan-cancer immunological analysis of bulk *FMNL1* mRNA expression

**Figure 2:**
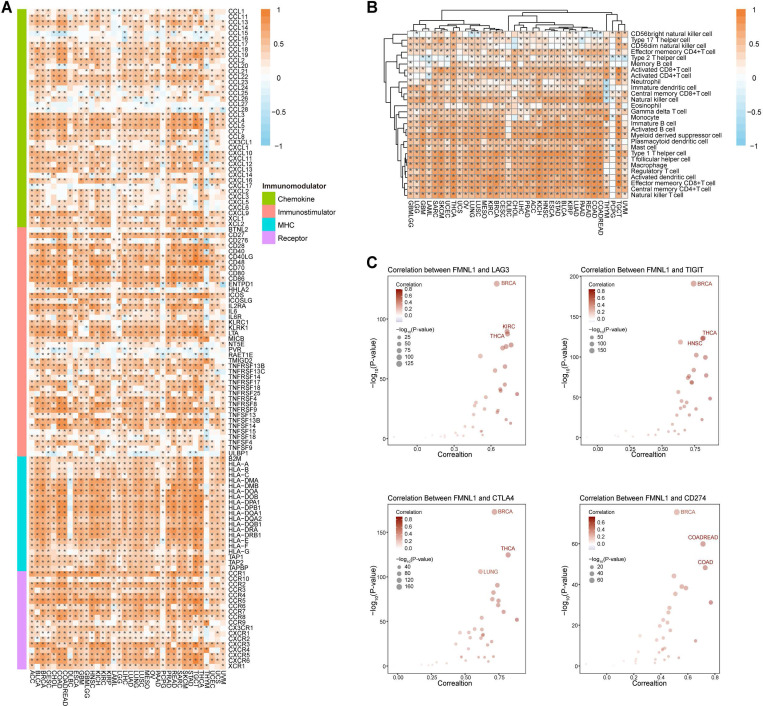
** Pan-cancer analysis of the immunological correlation of FMNL1 in the TCGA pan-cancer cohort.** (A) Correlations between FMNL1 and 122 immunomodulators (chemokines, immunostimulators, MHC, and receptors). The color indicates the correlation coefficient. The asterisks indicate significant differences assessed by Pearson analysis. (B) Correlations between FMNL1 and 28 TIICs calculated using the ssGSEA algorithm. The color indicates the correlation coefficient. The asterisks indicate significant differences assessed by Pearson analysis. (C) Correlation between FMNL1 and 4 immune checkpoints, LAG3, TIGIT, CTLA4, and CD274. The dots represent cancer types. The y-axis represents the Pearson correlation coefficient, while the x-axis represents -log_10_ (P-value).

Whether bulk *FMNL1* mRNA expression was correlated with the immuno-hot TME in pan-cancer was next assessed. As Figure 2A showed that FMNL1 was positively correlated with chemokines, immunostimulators, MHC molecules, and receptors in most types of cancer except PCPG. The FMNL1 expression was substantially linked with the majority of TIICs in pan-cancer, with the exception of PCPG, according to the results of the computation of TIICs in the TME using the ssGSEA method (Figure 2B). Additionally, the associations between FMNL1 and immune checkpoint expressions in various cancer types were examined, and the results were equally encouraging (Figure 2C).

**Figure 3:**
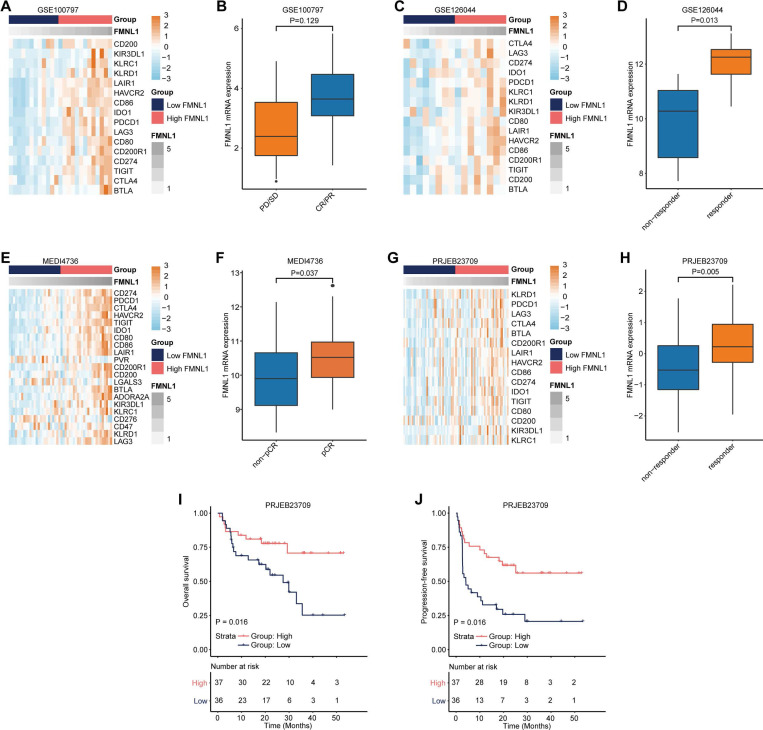
** Predictive value of FMNL1 in the response to immunotherapy.** (A, B) Correlations between FMNL1 and immune checkpoints expressions and predictive value of FMNL1 in the response to immunotherapy in the GSE100797 cohort. (C, D) Correlations between FMNL1 and immune checkpoints expressions and predictive value of FMNL1 in the response to immunotherapy in the GSE126044 cohort. (E, F) Correlations between FMNL1 and immune checkpoints expressions and predictive value of FMNL1 in the response to immunotherapy in the MEDI4736 cohort. (G, H) Correlations between FMNL1 and immune checkpoints expressions and predictive value of FMNL1 in the response to immunotherapy in the PRJEB23709 cohort. (I, J) Prognostic value of FMNL1 in the PRJEB23709 cohort in terms of OS and PFS.

As a matter of course, patients with high FMNL1 expression should exhibit better responses to immunotherapy due to the tight immunological correlations of FMNL1 in pan-cancer. We collected three datasets which included RNA-seq data from patients receiving immunotherapy. In the GSE100797, GSE126044, MEDI4736, and PRJEB23079 datasets, FMNL1 was also related to most immune checkpoints expressions (Figure 3A, 3C, 3E, 3G). In addition, FMNL1 was highly expressed in the patients with higher response to immunotherapy (Figure 3B, 3D, 3F, 3H). Moreover, patients with high FMNL1 expression exhibited prolonged OS and PFS in the PRJEB23079 dataset (Figure 3I-J). Collectively, these findings suggest that bulk *FMNL1* mRNA expression is a pan-cancer classifier that identifies immuno-hot tumors and forecasts immunotherapy response.

### Single-cell analysis and mQIF revealing FMNL1 is a biomarker for immune cells

**Figure 4:**
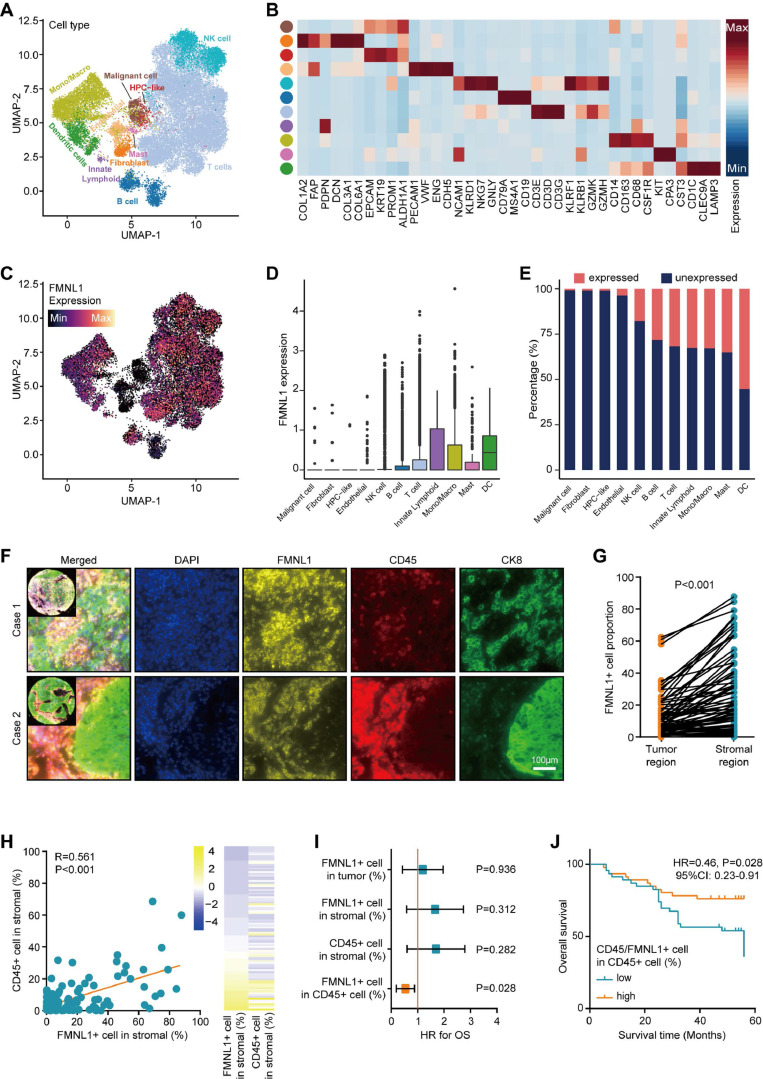
** Expression pattern of FMNL1 revealed by scRNA-seq and mQIF.** (A) UMAP visualization of eleven cell types in HCC patients in the merged dataset of GSE98638, GSE125449, and GSE140228. (B) Heatmap of the expression of cell type-specific genes which were reported in previous studies. (C) Expression levels of FMNL1 overlaid on the UMAP representation. (D) Boxplot displaying the expression levels of FMNL1 among cell types. (E) Stacked histogram showing the percentage of cells expressed FMNL1 (count > 0, pink) and unexpressed FMNL1 (count = 0, blue) in each cell type. (F) Representative images revealing FMNL1, CD15, and CK8 staining in HCC using mQIF in the HLivH180Su17 cohort. Magnification, 200×, Bar = 100μm. (G) Comparison of the positive rate of FMNL1 in the tumor and stromal regions. (H) Correlation between FMNL1 positive and CD45 positive rate in the stromal region in HCC. (I, J) The prognostic value of FMNL1 positive rate in the tumor region, FMNL1 positive rate in the stromal region, CD45 positive rate in the stromal region, and positive rate of FMNL1 in CD45^+^ cells in HCC.

Subsequently, the expression pattern of FMNL1 in HCC was investigated. The merged scRNA-seq data of HCC was utilized to describe the expression pattern of FMNL1 at the single-cell level (Figure 4A-B). As Figure 4C-E revealed, FMNL1 was highly expressed in various immune cells but lowly expression in tumor cells, HPC-like cells, fibroblasts, and endothelial cells. Considering that FMNL1 was expressed in various immune cells, we hypothesized that FMNL1 may be a pan-immune cells marker.

Then, mQIF was employed to validate the expression pattern of FMNL1 using co-staining of FMNL1, CD45 (a biomarker for most immune cells), and CK8 (a biomarker for glandular epithelium and adenocarcinoma cell). The clinical parameters of the in-house cohort were exhibited in Supplementary Table S2. The results showed that, FMNL1 was highly expressed and co-localized with CD45 in the stromal region (Figure 4F-H). Moreover, the prognostic value of FMNL1 in HCC was explored. As Figure 4I exhibited that, the positive rates of FMNL1 in the tumor region, FMNL1 in the stromal region, and CD45 in the stromal were not associated with prognosis. However, the positive rate of FMNL1 in CD45^+^ cells was associated with prolonged OS (Figure 4I-J). We also examined the correlation between FMNL1 expression and clinical parameters. The results were similar to the findings from the TCGA cohort, namely FMNL1 was correlated with gender (Supplementary Table S3).

To further explore the role of FMNL1 in formation of the inflamed tumor microenvironment (TME) in HCC patients, we firstly divided the immune subpopulations into FMNL1+ and FMNL1- based on the expressed count of FMNL1. Then, CellPhoneDB software was performed to dissect the interactions among various subpopulations. Results showed that compared with FMNL1- immune cells, FMNL1+ immune cells presented significantly more interactions with other cell subpopulations (Figure S2A-B). Notably, we calculated the difference in interaction numbers among T/NK (FMNL1+ and FMNL1-) and other subpopulations. Compared with T/NK (FMNL1-) cells, T/NK (FMNL1+) cells showed the highest communication strength with other T cells and malignant cells (Figure S2C), which potentially take part in the formation of an inflamed TME.

We further identified the significant ligand-receptor interactions among these cell types using CellphoneDB. Results showed that T/NK (FMNL1-) and myeloid (FMNL1-) cells rarely interacted with malignant cells, but T/NK (FMNL1+) and myeloid (FMNL1+) cells presented many unique interactions with malignant cells (Figure S2D-E).

**Figure 5:**
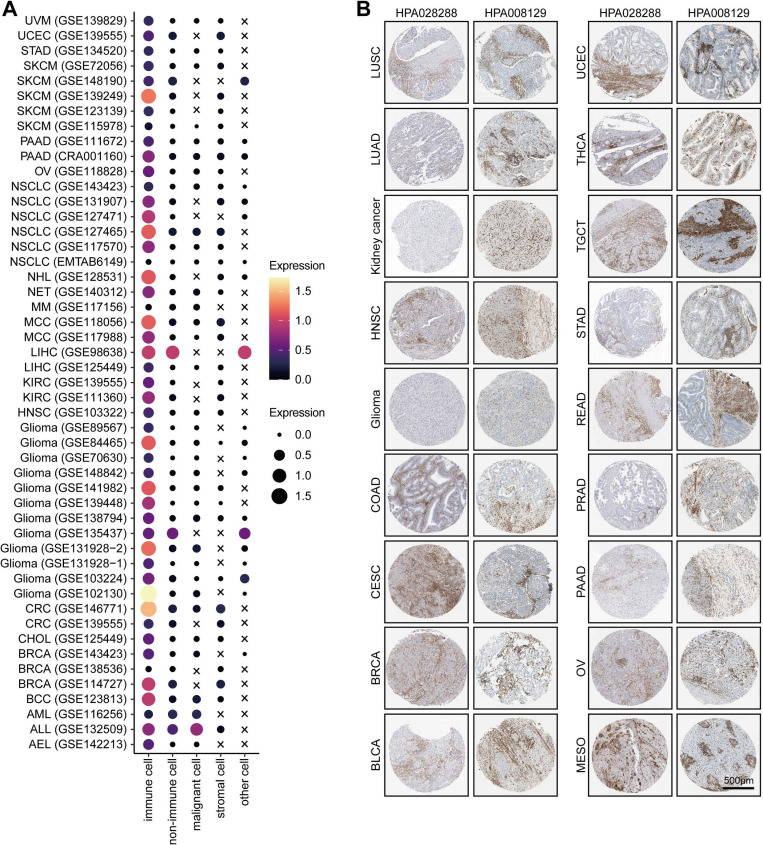
** Pan-cancer analysis of the expression pattern of FMNL1.** (A) Expression of FMNL1 at the single-cell level in multiple datasets. The original data was obtained from the TISCH database. (B) Immunohistochemistry staining revealed the expression of FMNL1 across cancer types. The original data was obtained from the HPA database.

For example, T/NK (FMNL1+) cells communicated with malignant cells via CCR6-CCL20 (Figure S2D), which involved in the recruitment of T/NK cells 40, 41 and promoted the invasion and metastasis of tumor cells 42. Specifically, our results showed that some inhibitory interactions, such as PVR-CD226, SPP1-CD44, and PDCD1-FAM3C interactions, were detected between malignant cells and T/NK (FMNL1+) cells (Figure S2D). Notably, a recent study found that the intercellular communications between PVR+ malignant cells and CD226+ T cells can enhance the anti-tumor immune response 43. In addition, malignant cells also had frequent interactions with myeloid (FMNL1+) cells via many ligand-receptor pairs, such as OSMR-OSM (Figure S2E), which took participate in the process and invasion of tumor cells 44-47. Besides, some famous inhibitory interactions, such as CD74-MIF, also found between malignant and myeloid (FMNL1+) cells (Figure S2E). Totally, based on the interactions among cell types, we explained the formation of the inflamed TME of patients with high FMNL1 expression, and further explored the unique and potential targeted ligand-receptor pairs between the T/NK (FMNL1+) / myeloid (FMNL1+) and malignant cells.Furthermore, we also explored the various expression of FMNL1 in different cell types in pan-cancer. Based on the data from the TISCH database, we revealed that FMNL1 was highly expressed in immune cells (Figure 5A). The immunohistochemistry data from the HPA database also supported that FMNL1 was highly expressed in the stromal region (Figure 5B). Overall, FMNL1 is a novel biomarker for immune cells, leading to the consequent immunological correlations in routine analysis using bulk RNA-seq data.

## Discussion

Recently, cancer immuno-correlation analysis of candidates is a boom in bioinformatics 2, 48, 49. However, some studies failed to draw more deeply meaningful conclusions just with superficial analysis. As an example of self-criticism, we revealed that FMNL1 was tightly related to immune infiltration in gastric cancer in our previous research 4, but no in-depth analysis was performed. In addition, genes expressed in tumor cells are preferred above those expressed in immune cells or other cells, as advised by a protocol to identify novel immunotherapy biomarkers. The expression of genes expressed in non-tumor cells may not be assessed due to the high purity of special tumors 23. In this research, based on public data and biological validation, we found that FMNL1 was just a novel biomarker for immune cells in HCC, but was not an immunologically correlated gene in tumor cells. Due to the specific expression pattern of FMNL1, its obvious correlation with immune TME could be observed in routine analysis based on bulk RNA-seq data, and its function act as a cancer immunologically correlated gene may be irrelevantly speculate.

FMNL1 is a classic member of Formin proteins and mediates the polymerization of F-actin 50. Given the significant role of FMNL1 in F-actin networks, FMNL1 is involved in podosome dynamics, phagocytosis, cell adhesion, and cell migration 51. According to published reports, the oncogenic role of FMNL1 has been preliminarily established. FMNL1 was elevated in multiple types of cancer and facilitated cell invasiveness 9, 52, 53. In terms of mechanisms, in addition to mediating cytoskeletal remodeling, FMNL1 could promote tumor cell aggressiveness through multiple mechanisms. In nasopharyngeal carcinoma, FMNL1 promoted cell aggressiveness by epigenetically up-regulating MTA1 8. In NSCLC, inhibition of FMNL1 could suppress bone metastasis *via* restraining TGF-β1 signaling 54. Hovever, the expression pattern of FMNL1 in cancer has not been well explored.

FMNL1 is mostly expressed in immune cells and tissues, including peripheral blood leukocytes, the spleen, and the thymus, while playing a crucial oncogenic role in numerous cancer types 6, 55, 56. Additionally, FMNL1 is strongly expressed in a number of hematological malignancies, such as non-Hodgkin's lymphomas, lymphoid and myeloid leukemias, and malignant lymphoid and myeloid cell lines 6, 55, 57. As we all know, the tumor mass is complex and consists of malignant as well as anti-tumor immune cells 58. As revealed in this research based on scRNA-seq and mQIF, FMNL1 is highly expressed in immune cells instead of tumor cells in HCC. In addition, several studies has uncovered that FMNL1 was highly expressed in TIICs in breast cancer 24, 59. Due to this specific expression pattern, FMNL1 mRNA expression in tumor tissues was largely sourced from TIICs from bulk RNA-seq analysis.

In this report, we revealed that higher FMNL1 expression could predict well clinical outcome in HCC. However, prior studies showed that elevated FMNL1 expression was linked to poor prognosis in a number of malignancies, including clear cell renal cell carcinoma 60, gastric cancer 4, and glioblastoma 9. According to previous research, FMNL1 promoted the migration of T cells and macrophages 61, 62. Simultaneously, based on high positive rate of FMNL1 in CD45^+^ cells predicting prolonged OS verified in this research, we speculated that FMNL1^+^ immune cells had stronger migration capability, increasing their propensity to act as anti-tumor roles. In addition, FMNL1 was also could be detected in tumor cells, which acted as a critical oncogene in multiple cancers. As a result, we hypothesized that varied prognostic characteristics in diverse malignancies were caused by the balance of FMNL1 expression in tumor and immune cells.

## Conclusions

Overall, the in-depth analysis beyond bulk RNA-sequencing was prepormed involving the immunological correlation and cell subpopulation transcriptomic pattern of FMNL1. Although FMNL1 was tightly correlated with immune infiltration in HCC, and even in pan-cancer, it was a novel biomarker for immune cells, but might not be a critical regulator in tumor cells to mediate tumor immunity due to the distinctive expression pattern of FMNL1. Thus, blind immune infiltration analysis based on bulk RNA-seq data should be further validated using scRNA-seq or mQIF for more deeply appropriate conclusions.

## Supplementary Material

Supplementary figures and tables.

## Abbreviations

RNA-seq: RNA-sequencing
TME: tumor microenvironment
FMNL1: Formin-like gene 1
TIIC: tumor-infiltrating immune cell
TCGA: the Cancer Genome Atlas
HCC: hepatocellular carcinoma
scRNA-seq: single-cell RNA-sequencing
mQIF: multiplexed quantitative immunofluorescence
OS: overall survival
DSS: disease-specific survival
DFS: disease-free survival
PFS: progression-free survival
MHC: major histocompatibility complex
ssGSEA: single-sample gene sets enrichment analysis

## References

[1] Ly D, Li Q, Navab R, Zeltz C, Fang L, Cabanero M (2021). Tumor-Associated Regulatory T Cell Expression of LAIR2 Is Prognostic in Lung Adenocarcinoma. Cancers (Basel).

[2] Yang Z, Tian H, Bie F, Xu J, Zhou Z, Yang J (2021). ERAP2 Is Associated With Immune Infiltration and Predicts Favorable Prognosis in SqCLC. Front Immunol.

[3] Kuksin M, Morel D, Aglave M, Danlos FX, Marabelle A, Zinovyev A (2021). Applications of single-cell and bulk RNA sequencing in onco-immunology. Eur J Cancer.

[4] Nie H, Mei J, Zhang Q, An F, Zhan Q (2020). Systematic Characterization of the Expression and Prognostic Values of Formin-Like Gene Family in Gastric Cancer. DNA Cell Biol.

[5] Gardberg M, Heuser VD, Iljin K, Kampf C, Uhlen M, Carpen O (2014). Characterization of Leukocyte Formin FMNL1 Expression in Human Tissues. The journal of histochemistry and cytochemistry: official journal of the Histochemistry Society.

[6] Favaro PM, de Souza Medina S, Traina F, Basseres DS, Costa FF, Saad ST (2003). Human leukocyte formin: a novel protein expressed in lymphoid malignancies and associated with Akt. Biochemical and biophysical research communications.

[7] Harris ES, Rouiller I, Hanein D, Higgs HN (2006). Mechanistic differences in actin bundling activity of two mammalian formins, FRL1 and mDia2. J Biol Chem.

[8] Chen WH, Cai MY, Zhang JX, Wang FW, Tang LQ, Liao YJ (2018). FMNL1 mediates nasopharyngeal carcinoma cell aggressiveness by epigenetically upregulating MTA1. Oncogene.

[9] Higa N, Shinsato Y, Kamil M, Hirano T, Takajo T, Shimokawa M (2019). Formin-like 1 (FMNL1) Is Associated with Glioblastoma Multiforme Mesenchymal Subtype and Independently Predicts Poor Prognosis. International journal of molecular sciences.

[10] Zheng C, Zheng L, Yoo JK, Guo H, Zhang Y, Guo X (2017). Landscape of Infiltrating T Cells in Liver Cancer Revealed by Single-Cell Sequencing. Cell.

[11] Ma L, Hernandez MO, Zhao Y, Mehta M, Tran B, Kelly M (2019). Tumor Cell Biodiversity Drives Microenvironmental Reprogramming in Liver Cancer. Cancer cell.

[12] Zhang Q, He Y, Luo N, Patel SJ, Han Y, Gao R (2019). Landscape and Dynamics of Single Immune Cells in Hepatocellular Carcinoma. Cell.

[13] Yoshihara K, Shahmoradgoli M, Martinez E, Vegesna R, Kim H, Torres-Garcia W (2013). Inferring tumour purity and stromal and immune cell admixture from expression data. Nat Commun.

[14] Charoentong P, Finotello F, Angelova M, Mayer C, Efremova M, Rieder D (2017). Pan-cancer Immunogenomic Analyses Reveal Genotype-Immunophenotype Relationships and Predictors of Response to Checkpoint Blockade. Cell Rep.

[15] Li T, Fu J, Zeng Z, Cohen D, Li J, Chen Q (2020). TIMER2.0 for analysis of tumor-infiltrating immune cells. Nucleic Acids Res.

[16] Racle J, de Jonge K, Baumgaertner P, Speiser DE, Gfeller D (2017). Simultaneous enumeration of cancer and immune cell types from bulk tumor gene expression data. Elife.

[17] Becht E, Giraldo NA, Lacroix L, Buttard B, Elarouci N, Petitprez F (2016). Estimating the population abundance of tissue-infiltrating immune and stromal cell populations using gene expression. Genome biology.

[18] Finotello F, Mayer C, Plattner C, Laschober G, Rieder D, Hackl H (2019). Molecular and pharmacological modulators of the tumor immune contexture revealed by deconvolution of RNA-seq data. Genome medicine.

[19] Ru B, Wong CN, Tong Y, Zhong JY, Zhong SSW, Wu WC (2019). TISIDB: an integrated repository portal for tumor-immune system interactions. Bioinformatics.

[20] Xu L, Deng C, Pang B, Zhang X, Liu W, Liao G (2018). TIP: A Web Server for Resolving Tumor Immunophenotype Profiling. Cancer Res.

[21] Bindea G, Mlecnik B, Tosolini M, Kirilovsky A, Waldner M, Obenauf AC (2013). Spatiotemporal dynamics of intratumoral immune cells reveal the immune landscape in human cancer. Immunity.

[22] Ayers M, Lunceford J, Nebozhyn M, Murphy E, Loboda A, Kaufman DR (2017). IFN-gamma-related mRNA profile predicts clinical response to PD-1 blockade. J Clin Invest.

[23] Mei J, Cai Y, Xu R, Zhu Y, Zhao X, Zhang Y (2023). Protocol to identify novel immunotherapy biomarkers based on transcriptomic data in human cancers. STAR Protocols.

[24] Mei J, Cai Y, Wang H, Xu R, Zhou J, Lu J Formin protein DIAPH1 positively regulates PD-L1 expression and predicts the therapeutic response to anti-PD-1/PD-L1 immunotherapy. Clinical Immunology. 2022:109204.

[25] Cai Y, Ji W, Sun C, Xu R, Chen X, Deng Y (2021). Interferon-Induced Transmembrane Protein 3 Shapes an Inflamed Tumor Microenvironment and Identifies Immuno-Hot Tumors. Front Immunol.

[26] Hu J, Yu A, Othmane B, Qiu D, Li H, Li C (2021). Siglec15 shapes a non-inflamed tumor microenvironment and predicts the molecular subtype in bladder cancer. Theranostics.

[27] Wolf FA, Angerer P, Theis FJ (2018). SCANPY: large-scale single-cell gene expression data analysis. Genome Biol.

[28] Polanski K, Young MD, Miao Z, Meyer KB, Teichmann SA, Park JE (2020). BBKNN: fast batch alignment of single cell transcriptomes. Bioinformatics.

[29] Traag VA, Waltman L, van Eck NJ (2019). From Louvain to Leiden: guaranteeing well-connected communities. Sci Rep.

[30] Becht E, McInnes L, Healy J, Dutertre CA, Kwok IWH, Ng LG (2019). Dimensionality reduction for visualizing single-cell data using UMAP. Nature biotechnology.

[31] Sun D, Wang J, Han Y, Dong X, Ge J, Zheng R (2021). TISCH: a comprehensive web resource enabling interactive single-cell transcriptome visualization of tumor microenvironment. Nucleic acids research.

[32] Efremova M, Vento-Tormo M, Teichmann SA, Vento-Tormo R (2020). CellPhoneDB: inferring cell-cell communication from combined expression of multi-subunit ligand-receptor complexes. Nat Protoc.

[33] Wang Y, Deng J, Wang L, Zhou T, Yang J, Tian Z (2020). Expression and clinical significance of PD-L1, B7-H3, B7-H4 and VISTA in craniopharyngioma. Journal for immunotherapy of cancer.

[34] Blenman KRM, Marczyk M, Karn T, Qing T, Li X, Gunasekharan V (2022). Predictive Markers of Response to Neoadjuvant Durvalumab with Nab-Paclitaxel and Dose-Dense Doxorubicin/Cyclophosphamide in Basal-Like Triple-Negative Breast Cancer. Clin Cancer Res.

[35] Lauss M, Donia M, Harbst K, Andersen R, Mitra S, Rosengren F (2017). Mutational and putative neoantigen load predict clinical benefit of adoptive T cell therapy in melanoma. Nat Commun.

[36] Cho JW, Hong MH, Ha SJ, Kim YJ, Cho BC, Lee I (2020). Genome-wide identification of differentially methylated promoters and enhancers associated with response to anti-PD-1 therapy in non-small cell lung cancer. Exp Mol Med.

[37] Gide TN, Quek C, Menzies AM, Tasker AT, Shang P, Holst J (2019). Distinct Immune Cell Populations Define Response to Anti-PD-1 Monotherapy and Anti-PD-1/Anti-CTLA-4 Combined Therapy. Cancer Cell.

[38] Uhlen M, Fagerberg L, Hallstrom BM, Lindskog C, Oksvold P, Mardinoglu A (2015). Proteomics. Tissue-based map of the human proteome. Science.

[39] Chen X, Chen H, Yao H, Zhao K, Zhang Y, He D (2021). Turning up the heat on non-immunoreactive tumors: pyroptosis influences the tumor immune microenvironment in bladder cancer. Oncogene.

[40] Chew V, Lai L, Pan L, Lim CJ, Li J, Ong R (2017). Delineation of an immunosuppressive gradient in hepatocellular carcinoma using high-dimensional proteomic and transcriptomic analyses. Proc Natl Acad Sci U S A.

[41] Chen KJ, Lin SZ, Zhou L, Xie HY, Zhou WH, Taki-Eldin A (2011). Selective recruitment of regulatory T cell through CCR6-CCL20 in hepatocellular carcinoma fosters tumor progression and predicts poor prognosis. PLoS One.

[42] Du D, Liu Y, Qian H, Zhang B, Tang X, Zhang T (2014). The effects of the CCR6/CCL20 biological axis on the invasion and metastasis of hepatocellular carcinoma. International journal of molecular sciences.

[43] Zhang H, Zhang Y, Dong J, Li B, Xu C, Wei M (2021). Recombinant oncolytic adenovirus expressing a soluble PVR elicits long-term antitumor immune surveillance. Mol Ther Oncolytics.

[44] Geethadevi A, Nair A, Parashar D, Ku Z, Xiong W, Deng H (2021). Oncostatin M Receptor-Targeted Antibodies Suppress STAT3 Signaling and Inhibit Ovarian Cancer Growth. Cancer Res.

[45] Parashar D, Geethadevi A, Aure MR, Mishra J, George J, Chen C (2019). miRNA551b-3p Activates an Oncostatin Signaling Module for the Progression of Triple-Negative Breast Cancer. Cell Rep.

[46] Hara T, Chanoch-Myers R, Mathewson ND, Myskiw C, Atta L, Bussema L (2021). Interactions between cancer cells and immune cells drive transitions to mesenchymal-like states in glioblastoma. Cancer Cell.

[47] Lee BY, Hogg EKJ, Below CR, Kononov A, Blanco-Gomez A, Heider F (2021). Heterocellular OSM-OSMR signalling reprograms fibroblasts to promote pancreatic cancer growth and metastasis. Nat Commun.

[48] Zhu ZY, Tang N, Wang MF, Zhou JC, Wang JL, Ren HZ (2021). Comprehensive Pan-Cancer Genomic Analysis Reveals PHF19 as a Carcinogenic Indicator Related to Immune Infiltration and Prognosis of Hepatocellular Carcinoma. Front Immunol.

[49] Yue Y, Zhang Q, Sun Z (2021). CX3CR1 Acts as a Protective Biomarker in the Tumor Microenvironment of Colorectal Cancer. Front Immunol.

[50] Esue O, Harris ES, Higgs HN, Wirtz D (2008). The filamentous actin cross-linking/bundling activity of mammalian formins. Journal of molecular biology.

[51] Mersich AT, Miller MR, Chkourko H, Blystone SD (2010). The formin FRL1 (FMNL1) is an essential component of macrophage podosomes. Cytoskeleton.

[52] Zhang MF, Li QL, Yang YF, Cao Y, Zhang CZ (2020). FMNL1 Exhibits Pro-Metastatic Activity via CXCR2 in Clear Cell Renal Cell Carcinoma. Front Oncol.

[53] Cui F, Ji Y, Wang M, Gao F, Li Y, Li X (2019). miR-143 inhibits proliferation and metastasis of nasopharyngeal carcinoma cells via targeting FMNL1 based on clinical and radiologic findings. J Cell Biochem.

[54] Yang XY, Liao JJ, Xue WR (2019). FMNL1 down-regulation suppresses bone metastasis through reducing TGF-beta1 expression in non-small cell lung cancer (NSCLC). Biomed Pharmacother.

[55] Schuster IG, Busch DH, Eppinger E, Kremmer E, Milosevic S, Hennard C (2007). Allorestricted T cells with specificity for the FMNL1-derived peptide PP2 have potent antitumor activity against hematologic and other malignancies. Blood.

[56] Han Y, Eppinger E, Schuster IG, Weigand LU, Liang X, Kremmer E (2009). Formin-like 1 (FMNL1) is regulated by N-terminal myristoylation and induces polarized membrane blebbing. J Biol Chem.

[57] Favaro PM, Traina F, Vassallo J, Brousset P, Delsol G, Costa FF (2006). High expression of FMNL1 protein in T non-Hodgkin's lymphomas. Leukemia research.

[58] Duan Q, Zhang H, Zheng J, Zhang L (2020). Turning Cold into Hot: Firing up the Tumor Microenvironment. Trends Cancer.

[59] Gao E, Wang X, Wang F, Deng S, Xia W, Wang R (2022). Systematic Characterization of Expression Patterns and Immunocorrelations of Formin-Like Genes in Breast Cancer. BioMed research international.

[60] Ma G, Wang Z, Liu J, Fu S, Zhang L, Zheng D (2021). Quantitative proteomic analysis reveals sophisticated metabolic alteration and identifies FMNL1 as a prognostic marker in clear cell renal cell carcinoma. Journal of Cancer.

[61] Miller MR, Blystone SD (2015). Human Macrophages Utilize the Podosome Formin FMNL1 for Adhesion and Migration. CellBio.

[62] Thompson SB, Sandor AM, Lui V, Chung JW, Waldman MM, Long RA (2020). Formin-like 1 mediates effector T cell trafficking to inflammatory sites to enable T cell-mediated autoimmunity. Elife.

