# Supplementary material for: Formin protein FMNL1 is a biomarker for tumor-infiltrating immune cells and associated with well immunotherapeutic response
Source: J Cancer. 2023 Sep 11;14(16):2978–89. doi: 10.7150/jca.86965 (PMC10583584; doi:10.7150/jca.86965)
Supplement: Supplementary file 1 — Supplementary figures and tables. [file jcav14p2978s1.pdf]

Supplementary figures

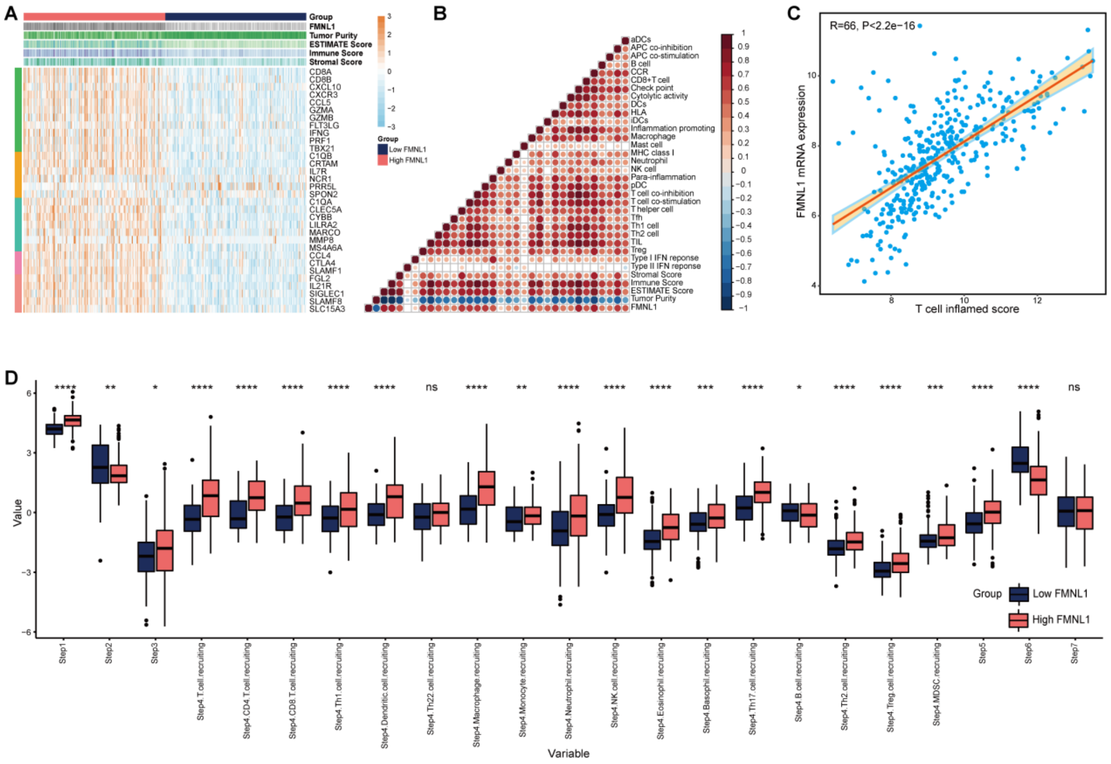

Figure S1. FMNL1 identifies the immuno-hot TME in HCC (supplementary)

(A) Expression levels of gene marker for immune cells between the high and low FMNL1 groups in HCC. (B) Correlation between FMNL1 and the infiltration levels of various immune cell populations and the activity of immune-related pathways calculated using the ssGSEA algorithm in HCC. The color reveals the Pearson correlation coefficient. (C) Correlation between FMNL1 and T cell inflamed score in HCC. (D) Differences in the various steps of the cancer immunity cycle calculated using the ssGSEA algorithm between the high and low FMNL1 groups in HCC.

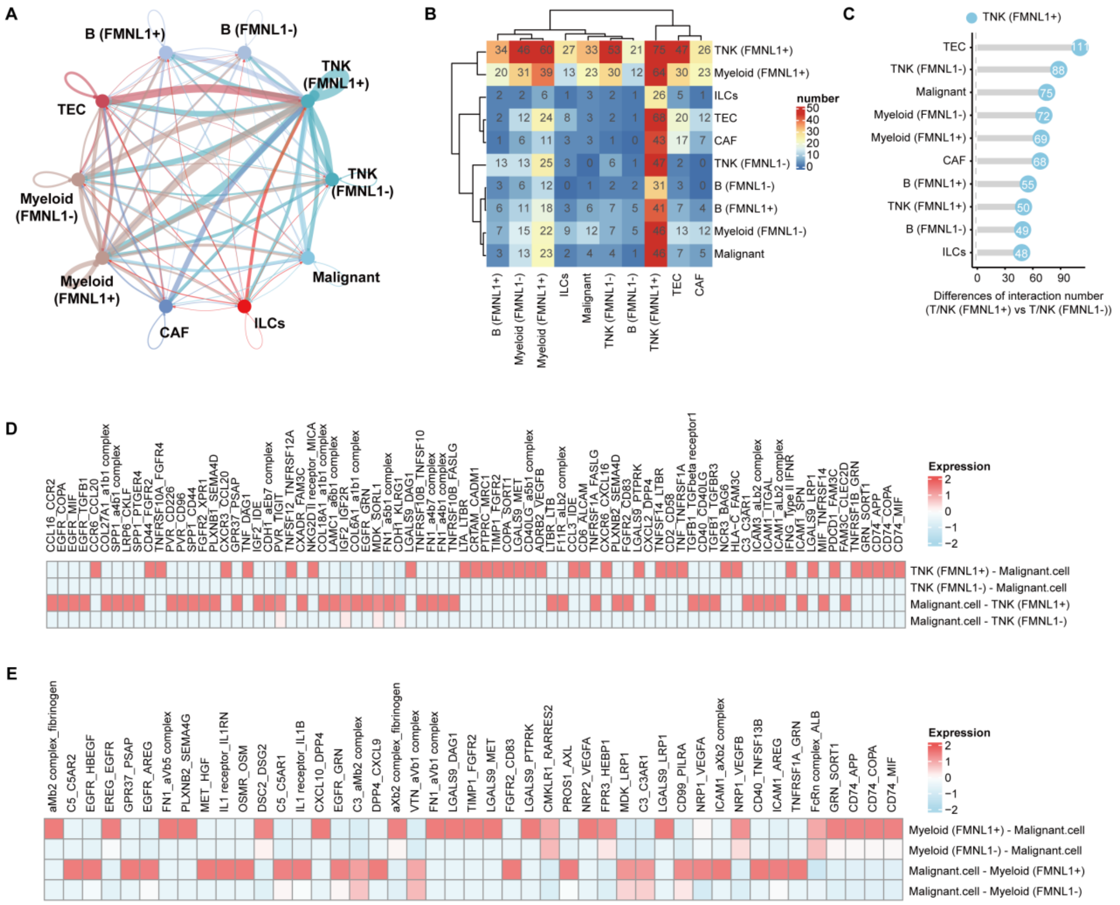

Figure S2. Cell-cell communications between cell subpopulations in the HCC scRNA-seq datasets.

(A-B) The interaction number of cell subpopulations in HCC patients. The thickness of the line represents the interaction number between the subpopulations estimated by CellPhoneDB. (C) The difference of the number of ligand-receptor interactions between T/NK (FMNL1+) and T/NK (FMNL1-). (D) Heatmap showing the specific ligand-receptor interactions between T/NK (FMNL1+) and malignant cells. (E) Heatmap showing the specific ligand-receptor interactions between myeloid (FMNL1+) and malignant cells.

Supplementary Table S1. Summary of abbreviations in the TCGA dataset.

Abbreviation	Full name
ACC	adrenocortical carcinoma
BLCA	bladder urothelial carcinoma
BRCA	breast invasive carcinoma
CESC	cervical & endocervical cancer
CHOL	cholangio carcinoma
COAD	colon adenocarcinoma
DLBC	lymphoid neoplasm diffuse large B-cell lymphoma
ESCA	esophageal carcinoma
GBM	glioblastoma multiforme
HNSC	head and neck squamous cell carcinoma
KICH	kidney chromophobe carcinoma
KIRC	kidney renal clear cell carcinoma
KIRP	kidney renal papillary cell carcinoma
LAML	acute myeloid leukemia
LGG	brain lower grade glioma
LIHC	liver hepatocellular carcinoma
LUAD	lung adenocarcinoma
LUSC	lung squamous cell carcinoma
MESO	mesothelioma
OV	ovarian serous cystadenocarcinoma
PAAD	pancreatic adenocarcinoma
PCPG	pheochromocytoma and paraganglioma
PRAD	prostate adenocarcinoma
READ	rectum adenocarcinoma
SARC	sarcoma
SKCM	skin cutaneous melanoma
STAD	stomach adenocarcinoma
TGCT	testicular germ cell tumors
THCA	thyroid carcinoma
THYM	thymoma
UCEC	uterine corpus endometrial carcinoma
UCS	uterine carcinosarcoma
UVM	uveal melanoma

Supplementary Table S2. Summary of clinical parameters of enrolled patients.

Clinical parameters	The TCGA cohort		The in-house cohort	
	number	%	number	%
gender				
male	252	32.44%	82	89.13%
female	121	67.56%	10	10.87%
age				
≤55	125	33.51%	59	64.13%
>55	247	66.22%	33	35.87%
unknown	1	0.27%		
grade				
well and medium	233	62.47%	45	48.91%
poor	135	36.19%	47	51.09%
unknown	5	1.34%	-	-
clinical stage				
1	172	46.11%	59	64.13%
2-4	177	47.45%	33	35.87%
unknown	24	6.43%	-	0.00%
case for OS analysis	367	98.39%	92	100.00%
case for DSS analysis	359	96.25%	-	-
case for DFS analysis	315	84.45%	-	-
case for PFS analysis	368	98.66%	-	-

Supplementary Table S3. Correlation between FMNL1 expression and clinical parameters of enrolled patients.

Clinical parameters	number	FMNL1 expression		P value
		low	high	
The TCGA cohort				
gender				
female	121	47	74	0.003
male	252	139	113	
age				
≤55	125	60	65	0.583
>55	247	126	121	
grade				
well and medium	233	119	114	0.589
poor	135	65	70	
clinical stage				
1	172	88	84	0.787
2-4	177	88	89	
The in-house cohort				
gender				
female	10	1	9	0.015
male	82	45	37	
age				
≤55	59	29	30	0.828
>55	33	17	16	
grade				
well and medium	45	22	23	0.835
poor	47	24	23	
clinical stage				
1	59	28	31	0.514
2-4	33	18	15	
